# Promoting Water Consumption on a Caribbean Island: An Intervention Using Children’s Social Networks at Schools

**DOI:** 10.3390/ijerph15040713

**Published:** 2018-04-10

**Authors:** Saskia C. M. Franken, Crystal R. Smit, Moniek Buijzen

**Affiliations:** 1 Faculty for Accounting, Finance and Marketing, University of Aruba, J.E. Irausquinplein 4, Oranjestad, Aruba; 2 Behavioural Science Institute, Radboud University, Montessorilaan 3, P.O. Box 9104, 6500 HE Nijmegen, The Netherlands; c.smit@bsi.ru.nl; m.buijzen@ru.nl

**Keywords:** Aruba, Caribbean, children, health, PROCESS moderation analysis, social norms, cluster randomized control trial, social network, sugar-sweetened beverage consumption, water consumption

## Abstract

Sugar-sweetened beverage (SSB) consumption and the associated childhood obesity are major concerns in the Caribbean, creating a need for interventions promoting water consumption as a healthy alternative. A social network-based intervention (SNI) was tested among Aruban children to increase their water consumption and behavioral intention to do so and, consequently, to decrease SSB consumption and the associated behavioral intention. In this study, the moderating effects of descriptive and injunctive norms were tested. A cluster randomized controlled trial was completed in schools (mean age = 11 years ± SD = 0.98; 54% girls). Children were assigned to the intervention group (IG; *n* = 192) or control group (CG; *n* = 185). IG children were exposed to peer influencers promoting water consumption and CG children were not. Regression analyses showed that water consumption increased for IG children with a high injunctive norm score (*p* = 0.05); however, their intention to consume more water remained unchanged (*p* = 0.42). Moreover, IG children showed a decrease in SSB consumption (*p* = 0.04) and an increase in their intention to consume less SSB (*p* = 0.00). These findings indicate that SNIs are a promising instrument for health behavioral changes for Aruba and other islands in the Caribbean region.

## 1. Introduction

The magnitude of the childhood obesity problem as a part of public health has been recognized worldwide [1] as well as in the Caribbean region [2,3,4,5,6,7,8]. This certainly applies to Aruba, an island in the Caribbean and a constituent country of the Kingdom of the Netherlands, where a large proportion (43%) of a sample of primary school-aged children were categorized overweight or obese [9]. An explanation might lie in the consumption of sugar-sweetened beverages (SSB) [10,11,12], which is the highest in the Caribbean region compared to 21 other world regions [13]. Caribbean-based behavioral health intervention studies among children are scarce and have not targeted beverage consumption behavior [14,15,16]. Furthermore, most intervention research on this topic has focused on the North American and European regions [13,17,18,19,20], so more research to investigate the effectiveness of interventions in other world regions is required. Given this, investigating an intervention program that aims to stimulate water consumption and, by doing so, reduce the consumption of SSB in a Caribbean context was deemed essential.

This study investigated the efficacy of an intervention that incorporates the social networks of Aruban children in their schools. The social network approach involves peer nominations to identify the most influential individuals in the peer network. We assumed that this approach would be appropriate for the Aruban context, given the island’s relatively small and closely connected social community [21]. Thus, the social environment may play a crucial influential role in such a setting. Several studies have found that social networks influence the consumption behavior of individuals [20,22,23,24]. An important explanation lies in individuals’ innate tendency to be sensitive to social norms they perceive from others in their network [24,25,26,27,28,29,30,31,32]. However, despite the acknowledged importance of the social environment, the underlying mechanism of social norms is unclear.

Social norms are defined as “implicit codes of conduct that provide a guide to appropriate action” [27]. Two types of social norms can be distinguished [33]. First, the “perceived descriptive norm” (PDN) refers to an individual’s perception of how most people behave and therefore consider these to be normal modeled behavior, for example, the extent to which peers consume water (PDNW) and SSB (PDNS). Second, the “perceived injunctive norm” (PIN) refers to an individual’s perception of other people’s beliefs regarding certain behavior, for example, the extent to which peers verbally approve of a child consuming water (PINW) or verbally disapprove of a child consuming SSB (PINS). Consequently, PDN and PIN present in social networks might moderate (i.e., interact with) the efficacy of a social network intervention. Thus, these norms may influence the change in the consumption behaviors of these children. Therefore, their roles were investigated in this study.

Thus, the objectives of this study were: (1) to test the efficacy of a social network-based intervention promoting water consumption in schools; and (2) to determine the degree to which PDN and PIN moderate the effect of the social network-based intervention. We hypothesized that children who are exposed to the social network-based intervention promoting water consumption would report an increase in their water consumption (H1a) and this effect would be stronger for children with a high PDNW (H1b) and high PINW (H1c; involving a positive PIN). The intervention would also result in a decrease in SSB consumption (H2a) and this effect would be stronger for children with a low PDNS (H2b) and a high PINS (H2c; involving a negative PIN). Furthermore, the expectation was that the children in the intervention group would have a greater intention to consume more water (H3a) and this effect would be stronger for children with a high PDNW (H3b) and high PINW (H3c). Additionally, children’s intention to consume less SSB would increase (H4a) and this effect would be stronger for children with a low PDNS (H4b) and a high PINS (H4c).

## 2. Materials and Methods

### 2.1. Design

The study was designed as a cluster randomized control trial, using the “Share H_2_O” intervention conducted by Smit et al. [20] in the Netherlands. The Share H_2_O intervention is based on social network principles, using influential peers to target primary school children’s water consumption behavior. The focus of the Share H_2_O intervention matched the objective of our study of improving children’s consumption behaviors. The intervention design involved two aspects: (1) identifying and training the most influential children as peer influencers (PIs) to promote water consumption and (2) asking the PIs to promote water consumption among children in their social networks at schools. All children completed the same pre- and post-intervention measures. A researcher unaffiliated with this study randomly allocated the participating schools into the intervention or control group. To avoid the risk of contamination of the intervention group by the control group in this relatively small island setting, randomization was partly restricted based on the location of the schools and the number of students per school. In the control group, no intervention occurred.

### 2.2. Participants

**Figure 1:**
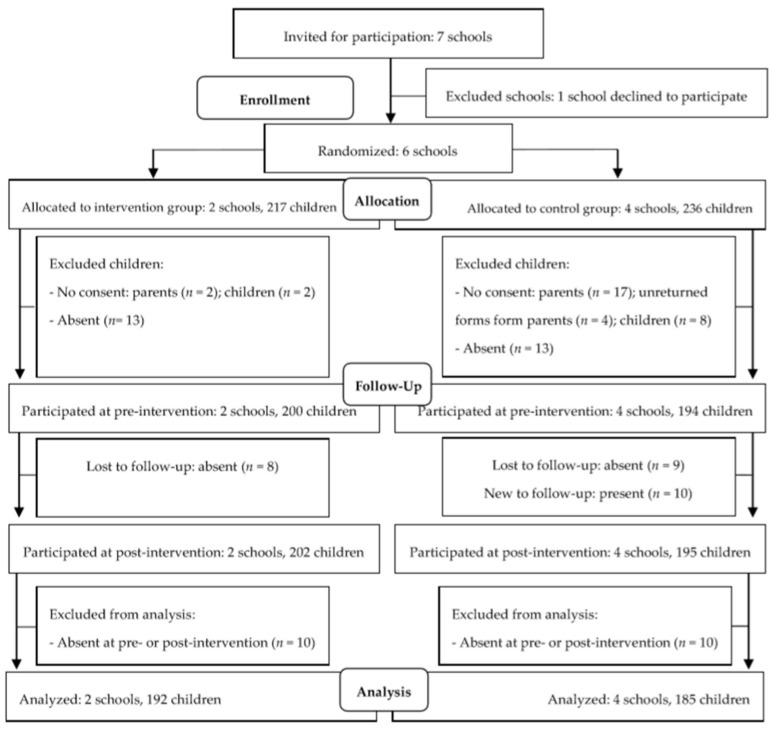
Flow diagram of participants.

Figure 1 displays the workflow used for recruitment, allocation, and number of participants in the intervention and control group at both measurements. The objective was to include 400 5th and 6th grade primary school children in the study. Therefore, six schools were approached to participate. One of these schools declined because of their involvement in another project, therefore, a seventh school was contacted. All the participating schools were not involved in any other health programs. After obtaining active informed consent from the head of the Aruban educational inspection, the schoolboard, and principals of the participating schools, randomization was performed. With permission from the principals and classroom-teachers, 453 parents received a letter with detailed information about the research project, giving them the opportunity to indicate whether or not they preferred their child to participate (passive informed consent). At the request of one principal, parents in that school in the control group had to provide active consent. Before handing out the questionnaires pre-intervention, children provided their active assent by signing a form indicating whether they would like to participate or not. The final sample for analysis included 377 children (54% girls) between 10 and 14 years old (mean = 11.4 ± SD = 0.98), with 192 in the intervention and 185 in the control group. Forty-two children in the intervention classes were selected as PIs and underwent the training program. 

### 2.3. Procedure

The intervention lasted eight weeks from January until March 2016. Before and after the intervention, children completed a paper-and-pencil questionnaire to determine their demographic information, consumption behavior, and behavioral intention to consume more water and less SSB. Data collectors primarily offered children the questionnaire in the official language, Papiamento. A small number of children (17) who lacked fluency in this language received a Dutch version, because this is the language used in Aruban educational settings. Following the study of Smit et al. [20], a selection procedure was used to identify the peer influencers (PIs) in the social networks at the schools using sociometric data provided by the children (see Section 2.5). These selected children were approached and then trained to become PIs to promote water consumption among their peers. To determine whether the children were aware of the purpose of the social network influence component of the project, they were asked during post-intervention measurement to describe the purpose of the study. Most children reported an association with water and SSB consumption; only the PIs were aware of the peer influencer component. The intervention procedure received approval by the Ethics Committee of the Faculty of Social Sciences at Radboud University: ECSW2014-1003-203. The present study was registered at the Netherlands Trial Registry: NTR5646.

### 2.4. Water Promotion Intervention Training

The purpose of the training was to provide the PIs the knowledge and skills to promote water consumption within their social networks in their schools. The training was offered by the first author during school hours and lasted approximately 90 min. The purposes of the training were: (1) to teach the PIs about the benefits of water consumption; (2) to motivate PIs to formulate their own arguments for consuming (more) water; (3) to teach PIs how to promote water consumption during peer interaction within their social networks; and (4) to encourage PIs to be an example to others by consuming (more) water in the proximity of other children in their social network. For this latter purpose, the PIs received a reusable water bottle. At the end of the training, the children were asked if they accepted their role of peer influencer, which all of them did. For a detailed description of the interactive elements and the theoretical foundations of the training, please refer to the publication of Smit et al. [20]. Furthermore, PIs received additional support by means of follow-up training sessions in weeks two and five of the intervention period. Support consisted of discussions about their experiences as PIs and to refresh the information that was shared during the training.

### 2.5. Measures

#### 2.5.1. Influential Peer Nominations

To identify PIs in the social networks in the intervention schools, the selection procedure was based on five sociometric questions that children answered during pre-intervention measurement. The children wrote down a maximum of five names of classmates whom they “respected,” “wanted to be like,” “looked up to,” “went for advice,” and “regarded as good leaders”. Of these nominations, 15% of the boys and 15% of the girls with the most nominations were invited to attend the training [20,23,34].

#### 2.5.2. Water and SSB Consumption

At pre- and post-intervention measurement, water consumption was measured by asking children how much water (0 = zero glasses to 8 = eight glasses) they drank on a normal school day and weekend day [20,35]. To facilitate the estimation of number of glasses, the children were told that a glass represents a can, a bottle, or a package. A total score for water consumption was constructed by averaging the school day and weekend day items, which demonstrated acceptable internal consistency (Spearman–Brown_pre_ = 0.68; Spearman–Brown_post_ = 0.66). At pre- and post-intervention measurement, SSB consumption was measured by asking how many glasses of juice, soft drinks, and energy and sport drinks they consumed (0 = zero glasses to 8 = eight glasses) on a normal school day and weekend day [20,35]. Each category had examples of brands or names for types of SSB. A total score for SSB consumption was constructed by calculating an average of the sum of the school day items and the sum of the weekend day items. The six items demonstrated good internal consistency (Cronbach’s alpha_pre_ = 0.77; Cronbach’s alpha_post_ = 0.75).

#### 2.5.3. Water and SSB Consumption Intention

Children’s intention to consume more water was measured by asking them the following three questions at both time-points: “Do you intend to drink more water?”, “Do you intend to drink more water during the next month?”, and “Do you intend to drink more water during the next year?” (1 = no, definitely not; 2 = no, I do not think so; 3 = yes, I think so; 4 = yes, definitely so) [20,36]. These items were averaged to create a water consumption intention scale, which demonstrated good internal consistency (Cronbach’s alpha_pre_ = 0.75; Cronbach’s alpha_post_ = 0.82). Behavioral intention to consume less SSB was measured at both time points by asking the same three questions (e.g., “Do you intend to drink less sugar-sweetened beverages?”). These items were averaged to create an SSB consumption intention scale, which demonstrated a good internal consistency (Cronbach’s alpha_pre_ = 0.85; Cronbach’s alpha_post_ = 0.88).

#### 2.5.4. Perceived Descriptive Norm Related to Water Consumption and SSB Consumption

The moderators, PDNW and PDNS, were measured by asking the children how often their friends consumed water and SSB [37]. The response categories for these four questions ranged from 1 = never; 2 = a few times; 3 = many times; to 4 = always. 

#### 2.5.5. Perceived Injunctive Norm Related to Water Consumption and SSB Consumption 

The moderators, PINW and PINS, were measured by asking children how often their friends approved of them consuming water and how often their friends disapproved of them consuming SSB [37]. The response categories for these four questions ranged from 1 = never; 2 = a few times; 3 = many times; to 4 = always. 

#### 2.5.6. Thirst Level

To control for individual differences, the children were presented with a 15 cm visual analogue scale (VAS) to measure the extent to which they felt thirsty before filling out the questions [38,39]. The scale ranged from 0 = not thirsty at all to 15 = very thirsty.

### 2.6. Statistical Analysis

Before conducting the main analyses to test the hypotheses, several preparatory analyses were conducted. First, we assessed skewness and kurtosis to confirm the normal distribution of the dependent variables. Second, a randomization check was conducted by means of independent samples *t*-test and Pearson’s chi square test to assess whether the randomization resulted in a balanced distribution across the intervention and control group. Third, to determine whether thirst, sex, and age were correlated with the dependent and moderating variables, Pearson’s correlation analyses were conducted. Then, for the main analyses, we conducted linear regression analyses for water and SSB consumption behavior, for water and SSB behavioral consumption intention, and for PDNW, PINW, PDNS, and PINS by using PROCESS (SPSS version 2.16.3, SPSS Inc., Chicago, IL, USA) developed by Hayes [40]. This SPSS macro centered the variables for the intervention group, PDNW, PINW, PDNS, and PINS before running the analyses. Significant interaction effects were further interpreted using simple slopes analysis, to interpret the effect of the intervention on children with a low degree of PDN or PIN (1 standard deviation (SD) below the mean) versus children with a high degree (1 SD above the mean) for each dependent variable. Finally, to determine the effect of training on the consumption behavior and behavioral consumption intention of the PIs themselves, paired sample *t*-tests were conducted as additional analyses. All analyses were run using SPSS version 24 (SPSS, Inc., Chicago, IL, USA). All tests were considered statistically significant at *p* ≤ 0.05.

## 3. Results

### 3.1. Preparatory Analyses

Skewness and kurtosis tests led to transforming the SSB consumption variable. The randomization analyses for the variables at pre-intervention demonstrated no differences (*p* > 0.05) between the intervention and the control groups, except for water consumption, PDNW, and PINW. Because these differences may have consequences for the main analyses, standardized *z*-values were created and included in the analyses instead of the raw values. The means and SDs for all variables at pre-intervention for the two groups are summarized in Table 1.

With the four dependent and the four moderating variables in the overall sample, Pearson’s correlation analyses showed that they were significantly correlated with thirst, sex, and age at the pre-intervention and/or at post-intervention measurements. Thirst was significantly correlated with water consumption (*r*_pre_ = 0.13, *p* = 0.01; *r*_post_ = 0.18, *p* = 0.00), but not with post-intervention water consumption intention (*r*_pre_ = 0.13, *p* = 0.01; *r*_post_ = 0.08, *p* = 0.14), nor with SSB consumption (*r*_pre_ = 0.07, *p* = 0.18; *r*_post_ = 0.03, *p* = 0.54). Furthermore, thirst was significantly correlated with post-intervention SSB consumption intention (*r*_pre_ = 0.03, *p* = 0.57; *r*_post_ = 0.13, *p* = 0.02), but not with any of the moderating variables. Sex was not significantly correlated with water consumption (*r*_pre_ = −0.07, *p* = 0.16; *r*_post_ = −0.05, *p* = 0.33), but was correlated with post-intervention SSB consumption (*r*_pre_ = −0.05, *p* = 0.30; *r*_post_ = −0.11, *p* = 0.04). In addition, sex was correlated with the behavioral intention to consume more water (*r*_pre_ = 0.08, *p* = 0.12; *r*_post_ = 0.13, *p* = 0.02), and with the intention to consume less SSB post-intervention (*r*_pre_ = 0.09, *p* = 0.07; *r*_post_ = 0.13, *p* = 0.01), and with PINW pre-intervention (*r*_pre_ = 0.12, *p* = 0.02; *r*_post_ = 0.05, *p* = 0.30), but not with the other moderators. Age was significantly correlated with post intervention water consumption (*r*_pre_ = −0.00, *p* = 0.97; *r*_post_ = −0.11, *p* = 0.04), but not with SSB consumption (*r*_pre_ = −0.02, *p* = 0.70; *r*_post_ = −0.02, *p* = 0.71). Age was also correlated with the behavioral intention to consume more water post-intervention (*r*_pre_ = −0.08, *p* = 0.12; *r*_post_ = −0.11, *p* = 0.04) and with the behavioral intention to consume less SSB (*r*_pre_ = −0.10, *p* = 0.05; *r*_post_ = −0.13, *p* = 0.02), but was not correlated with any of the moderating variables. Because thirst, sex, and age were correlated with certain dependent and moderating variables at certain time points, they were included as covariates in the analyses to ensure they did not confound the effect.

### 3.2. Main Analyses

#### 3.2.1. Water and SSB Consumption

**Figure 2:**
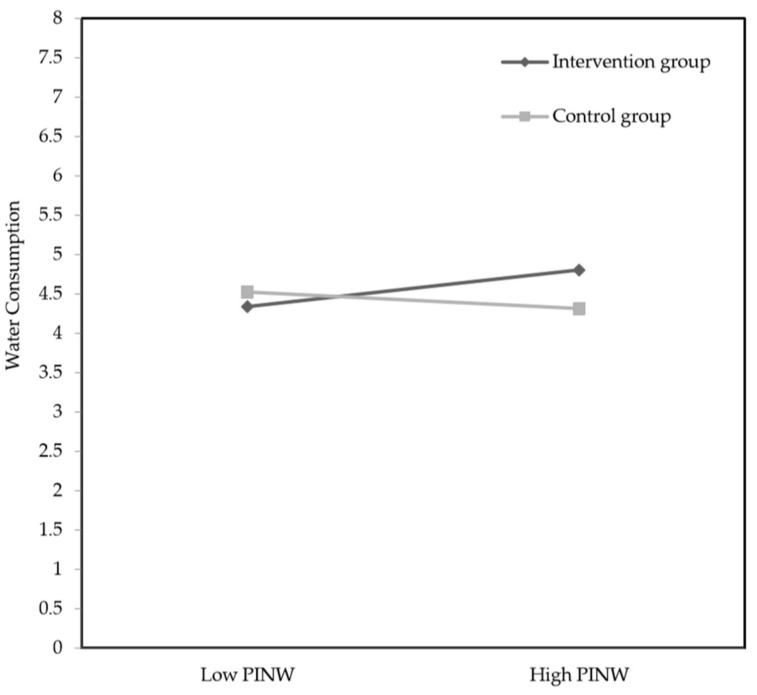
Interaction effects between the intervention group or control group and low or high perceived injunctive norm related to water consumption (PINW) for post-intervention water consumption.

Table 2 and Table 3 show the results of the efficacy of the intervention and moderators on water consumption and SSB consumption, respectively. For water consumption, the main effect of the intervention was nonsignificant (*p* = 0.50). The interaction term for the intervention and PINW was significant (*p* = 0.03). Further interpretation of the simple slopes of this interaction revealed that the intervention was significant for children in the intervention group with high PINW (*β* = 0.49; SE = 0.25; *t* = 2.00; *p* = 0.05; 95% CI: 0.01, 0.97), but not for children with a low PINW (*β* = −0.19; SE = 0.24; *t* = −0.77; *p* = 0.44; 95% CI: −0.66, 0.29; Figure 2). This significant interaction meant that after the intervention period, children with high PINW in the intervention group drank 0.50 units (i.e., glasses) more than children in the control group with high PINW (mean 4.81 and mean 4.31, respectively). In addition, after the intervention period, children with low PINW in the intervention group drank less units of water than children in the control group with low PINW (see Figure 2), but this difference of 0.18 units was not significant (mean 4.34 and mean 4.52, respectively). For SSB consumption (Table 3), the main effect of the intervention was significant (*p* = 0.04). PDNS and PINS did not moderate the effect of the intervention on SSB consumption. This result meant that after the intervention period, children in the intervention group consumed 0.14 units (i.e., glasses) less SSB compared to children in the control group (mean 4.80 and mean 4.94, respectively). Thus, these results indicate that the intervention significantly increased water consumption among children with a high PINW. Furthermore, the intervention significantly reduced SSB consumption for the children in the intervention group, as compared to the control group. Hence, these findings support H1c and H2a and do not support H1a, H1b, H2b, and H2c.

#### 3.2.2. Water and SSB Consumption Intention

Table 4 and Table 5 show the results of the effect of the intervention and the moderators on the behavioral consumption intention to consume more water and less SSB, respectively. The intervention had no significant effect on the behavioral intention to consume more water (*p* = 0.42), nor was this effect moderated by PDNW or PINW. For the intention to consume less SSB, the effect of the intervention was significant (*p* = 0.00), but was not moderated by PDNS or PINS. This entailed that after the intervention period, children in the intervention group had a 0.35 greater intention-score to consume less SSB than children in the control group (mean 3.08 and mean 2.73, respectively). These findings indicate that the intervention did not affect the intention to consume more water, but did increase children’s intention to consume less SSB. Furthermore, the effect of the intervention on both these dependent variables was not moderated by social norms. Accordingly, these findings supported H4a and not H3a, H3b, H3c, H4b, or H4c. 

### 3.3. Additional Analyses

#### Water and SSB Consumption Behavior and Behavioral Consumption Intention of Peer Influencers

The paired sample *t*-test of the consumption behavior and behavioral consumption intention of the PIs (*n* = 41) revealed similar patterns to the main analysis. On average, the PIs showed an increase in water consumption (mean_pre_ = 4.56, SE = 0.30; mean_post_ = 4.94, SE = 0.27) and a decrease in SSB consumption (mean_pre_ = 4.67, SE = 0.41; mean_post_ = 3.96, *SE* = 0.37). The change in water consumption was not significant (*t*(40) = −1.15, *p* = 0.26), but was significant for SSB consumption (*t*(40) = 2.10, *p* = 0.04). The intervention had a significant positive effect on PIs’ intention to consume more water (mean_pre_ = 3.46, SE = 0.09; mean_post_ = 3.65, SE = 0.11; *t*(40) = −2.48; *p* = 0.02), and a marginally significant positive effect on their intention to consume less SSB (mean_pre_ = 3.02, SE = 0.12; mean_post_ = 3.31, SE = 0.12; *t*(40) = −1.93; *p* = 0.06).

## 4. Discussion

Given the importance of SSB consumption in childhood obesity, water consumption intervention is needed worldwide, and especially in the Caribbean region [10,11,12,13,16]. However, intervention research focusing on increasing water consumption to combat obesity has focused primarily on North America and Europe [13,17,18,19]. Therefore, the present study was conducted in the Caribbean, investigating the efficacy of a social network-based intervention wherein influential children in primary school classrooms promoted water consumption among their peers. Results showed that the intervention increased water consumption, but only for children who felt that their peers thought they should drink more water (i.e., perceived injunctive norm). Children’s intention to consume more water remained unchanged. In addition, the intervention led to a reduction in SSB consumption and an increase in intentions to consume less SSB. The study highlights the important role of the social environment, demonstrating that children who are sensitive to what their peers think about the behavior targeted in the intervention are more susceptible to the impacts of the social network-based interventions.

Notably, we used the exact same intervention that was conducted in the Netherlands, allowing a comparison of the outcomes between the two countries. In both studies, the findings indicate that the intervention positively changed water and SSB consumption. However, the impact was contingent on the perceived injunctive norm in the present study, indicating an effect in Aruba that is weaker compared to the Netherlands [20]. A possible explanation might lie in Aruba being situated in a tropical climate, which may naturally spur children to frequently consume water, making the encouragement to consume more water less applicable to Aruban children. This may be different for the Dutch target group, because they live in a temperate climate and may therefore be less naturally inclined to consume water. Of course, this assumed ceiling effect remains speculative and further research is needed for decisive conclusions.

Similar to the Dutch intervention and consistent with our expectations, this Caribbean intervention reduced SSB consumption. Moreover, children indicated a greater intent to consume less SSB. This finding is in line with previous studies showing that a constructive direct focus on a single behavior with positive health consequences can indirectly affect associated unhealthy behaviors [20,41,42,43]. For water-promoting interventions, this may imply that children understand the implicitly promoted message that SSB consumption is in fact unhealthy. This study demonstrates that this indirect mechanism holds true for social network-based interventions.

Some strengths, limitations, and suggestions for future research should be considered in the interpretation of our findings. To the best of our knowledge, this study was the first to determine the role of perceived social norms in a social network-based intervention. Our findings indicate that perceived social norms can moderate the efficacy of the intervention, dependent on the type of social norm and type of behavior in question. This is in line with the general assumption in the literature that perceived social norms affect food and beverage consumption [20,24,25,26,27,28,29,30,31,32]. However, further research is needed to pin-point the components of the PI-training that lead to which types of changes in perceived descriptive and injunctive norms, and how to best measure these mechanisms to clarify what changes PIs activate in their social environments regarding beverage consumption behaviors. Another strength of this study is that it is one of the few scientifically conducted interventions targeting children’s health related behaviors in the Caribbean region. Therefore, this study may serve as a regional benchmark for designing future interventions. Furthermore, the statistically significant effects for consumption behaviors and behavioral intentions involved relatively small differences in units and scores; however, even a small behavioral change and its corresponding caloric change per day could have health consequences over time. For example, a 12-oz serving of SSB contains about 10 teaspoons of sugar (140–150 calories) and eliminating this daily consumption or having the intention to eliminate it would contribute to preventing a child from gaining weight [10]. In addition, by adopting an existing intervention, the outcomes of both studies could be compared so we were therefore able to confirm that a social network-based approach is successful in changing water and SSB consumption behaviors. 

A limitation of this study is that children self-reported their behaviors and results may have been different if water consumption was measured directly, for example, by means of observations at school or using flow meters attached to the schools’ water fountains [20,44]. Furthermore, due to the social network-based approach, an unavoidable limitation of this study is that individual participants could not be randomized across conditions. Inherent to the principles of social network-based intervention, the only way to execute the study was using cluster randomization. Additionally, we did not measure the long-term effects of the intervention. Future research could investigate long-term effects, including changes in children’s weight. Furthermore, future research could consider other significant people in the social environment, such as parents, who play a role in children’s dietary behaviors [37,45]. To gain a better understanding of children’s beverage consumption behavior and to design intervention studies in the Caribbean, future research could examine the role of demographic factors and other behavior related factors, such as attitude and motivation regarding the consumption of water and SSB, which have been shown to be important predictors of consumption behavior [10,29,36,46].

## 5. Conclusions

This study highlights the promising role of incorporating the influence of social networks in interventions when promoting healthy beverage consumption behavior among children in Aruba and, possibly, for other Caribbean islands. In addition, the role of social norms on children’s water consumption behavior was highlighted. This research shows that a constructive focus on a single positive behavior can lead to significant changes in unhealthy behaviors. The findings fill a gap in the existing knowledge about Aruba in the field of children’s water and SSB consumption behavior. However, in its entirety, the Caribbean region needs more research attention to determine how to promote the essential consumption of water.

## Figures and Tables

**Table 1:** Descriptive statistics for the intervention and control groups pre-intervention ^1,2,3^.

Measure	Intervention (*n* = 192)	Control (*n* = 185)	*p* ^4^
Age (years)	11.4 ± 0.9 (10–14)	11.4 ± 1.0 (10–14)	0.72
Boys/girls (*n/n*)	93/99	82/103	0.42
Thirst (15-cm Visual Analogue Scale)	5.8 ± 4.4 (0–15)	5.9 ± 4.7 (0–15)	0.83
Water consumption (i.e., glasses)	4.7 ± 1.9 (1–8)	4.3 ± 1.9 (1–8)	0.02
SSB ^5^ consumption (i.e., glasses)	5.2 ± 3.5 (0–20)	4.9 ± 3.0 (0–16)	0.27
Water consumption intention	3.4 ± 0.6 (1–4)	3.4 ± 0.7 (1–4)	0.75
SSB consumption intention	2.9 ± 0.8 (1–4)	2.9 ± 0.8 (1–4)	0.40
PDNW ^6^	2.8 ± 0.7 (1–4)	2.6 ± 0.8 (1–4)	0.04
PINW ^7^	2.7 ± 1.0 (1–4)	2.4 ± 1.0 (1–4)	0.00
PDNS ^8^	2.8 ± 0.7 (1–4)	2.9 ± 0.7 (2–4)	0.06
PINS ^9^	1.8 ± 0.8 (1–4)	1.6 ± 0.8 (1–4)	0.22

^1^ *n* = 377. ^2^ Values are presented as means ± standard deviations. ^3^ Values in parentheses denote the minimum and maximum of the response categories. ^4^ *p* values reflect the differences in the means between the two groups by independent samples *t*-test or Pearson’s chi square test. ^5^ Sugar-sweetened beverage. ^6^ Perceived descriptive norm related to water consumption. ^7^ Perceived injunctive norm related to water consumption. ^8^ Perceived descriptive norm related to SSB consumption. ^9^ Perceived injunctive norm related to SSB consumption. SSB: Sugar-sweetened beverage.

**Table 2:** Moderated regression analysis examining the interaction effects between intervention group, perceived descriptive norm related to water consumption (PDNW) and perceived injunctive norm related to water consumption (PINW) on water consumption over time ^1^.

Water Consumption T2 (*n* = 342)						
Variable	*β*	SE *β*	*t*	*p*	95% CI	
					Lower	Upper
Constant	6.96	1.08	6.45	0.00	4.84	9.08
Intervention (predictor)	0.12	0.18	0.67	0.50	−0.23	0.47
PDNW T1 (moderator)	0.03	0.09	0.34	0.74	−0.15	0.22
PINW T1 (moderator)	0.06	0.09	0.75	0.45	−0.11	0.24
Intervention X PDNW (interaction term)	−0.13	0.18	−0.70	0.49	−0.48	0.23
Intervention X PINW (interaction term)	0.38	0.17	2.20	0.03	0.04	0.71
Water consumption T1 (covariate)	1.01	0.09	10.81	0.00	0.83	1.19
Thirst T1 (covariate)	0.01	0.02	0.45	0.65	−0.04	0.06
Thirst T2 (covariate)	0.05	0.02	2.34	0.02	0.01	0.10
Sex (covariate)	−0.07	0.19	−0.37	0.71	−0.43	0.30
Age (covariate)	−0.24	0.09	−2.62	0.01	−0.42	−0.06
Overall model: *F*(10, 331) = 17.17, *p* = 0.00, *R*^2^ = 0.35						

^1^ T1 = pre-intervention and T2 = post-intervention.

**Table 3:** Moderated regression analysis examining the interaction effects between intervention group, perceived descriptive norm related to SSB consumption (PDNS) and perceived injunctive norm related to SSB consumption (PINS) on sugar-sweetened beverage (SSB) consumption over time ^1^.

SSB Consumption T2 (*n* = 350)						
Variable	*β*	SE *β*	*t*	*p*	95% CI	
					Lower	Upper
Constant	0.95	0.38	2.49	0.01	0.20	1.71
Intervention (predictor)	−0.12	0.06	−2.02	0.04	−0.24	0.00
PDNS T1 (moderator)	0.01	0.05	0.20	0.84	−0.08	0.10
PINS T1 (moderator)	0.05	0.04	1.30	0.19	−0.03	0.13
Intervention X PDNS (interaction term)	−0.04	0.09	−0.45	0.65	−0.22	0.14
Intervention X PINS (interaction term)	−0.08	0.08	−1.00	0.32	−0.23	0.08
SSB consumption T1 (covariate)	0.61	0.05	11.79	0.00	0.51	0.72
Thirst T1 (covariate)	−0.01	0.01	−1.07	0.28	−0.02	0.01
Thirst T2 (covariate)	0.01	0.01	0.98	0.33	−0.01	0.02
Sex (covariate)	−0.11	0.06	−1.69	0.09	−0.24	0.02
Age (covariate)	0.00	0.03	−0.05	0.96	−0.06	0.06
Overall model: *F*(10, 339) = 17.02, *p* = 0.00, *R*^2^ = 0.36						

^1^ T1 = pre-intervention and T2 = post-intervention.

**Table 4:** Moderated regression analysis examining the interaction effects between the intervention group, perceived descriptive norm related to water consumption (PDNW) and the perceived injunctive norm related to water consumption (PINW) on the behavioral intention to consume more water over time ^1^.

Behavioral Intention to Consume More Water T2 (*n* = 342)						
Variable	*β*	SE *β*	*t*	*p*	95% CI	
					Lower	Upper
Constant	1.97	0.45	4.42	0.00	1.09	2.84
Intervention (predictor)	0.05	0.06	0.80	0.42	−0.07	0.17
PDNW T1 (moderator)	0.07	0.04	1.84	0.07	−0.00	0.14
PINW T1 (moderator)	0.00	0.03	0.14	0.89	−0.06	0.07
Intervention X PDNW (interaction term)	−0.04	0.07	−0.61	0.54	−0.18	0.10
Intervention X PINW (interaction term)	−0.04	0.07	−0.54	0.59	−0.18	0.10
Behavioral intention to consume more water T1 (covariate)	0.57	0.06	9.25	0.00	0.45	0.69
Thirst T1 (covariate)	−0.00	0.01	−0.60	0.55	−0.02	0.01
Thirst T2 (covariate)	0.01	0.01	0.92	0.36	−0.01	0.02
Sex (covariate)	0.16	0.07	2.37	0.02	0.03	0.29
Age (covariate)	−0.06	0.03	−2.06	0.04	−0.13	0.00
Overall model: *F*(10, 331) = 13.06, *p* = 0.00, *R*^2^ = 0.34						

^1^ T1 = pre-intervention and T2 = post-intervention.

**Table 5:** Moderated regression analysis examining the interaction effects between intervention group, perceived descriptive norm related to SSB consumption (PDNS) and perceived injunctive norm related to SSB consumption (PINS) on the behavioral intention to consume less sugar-sweetened beverage (SSB) over time ^1^.

Behavioral Intention to Consume Less SSB T2 (*n* = 346)						
Variable	*β*	SE *β*	*t*	*p*	95% CI	
					Lower	Upper
Constant	1.91	0.51	3.74	0.00	0.91	2.91
Intervention (predictor)	0.30	0.08	3.93	0.00	0.15	0.46
PDNS T1 (moderator)	0.00	0.05	0.02	0.98	−0.10	0.10
PINS T1 (moderator)	0.11	0.05	2.16	0.03	0.01	0.20
Intervention X PDNS (interaction term)	−0.11	0.11	−1.04	0.30	−0.33	0.10
Intervention X PINS (interaction term)	0.14	0.10	1.46	0.14	−0.05	0.33
Behavioral intention to consume less SSB T1 (covariate)	0.46	0.06	7.66	0.00	0.35	0.58
Thirst T1 (covariate)	−0.01	0.01	−0.73	0.47	−0.03	0.01
Thirst T2 (covariate)	0.02	0.01	2.37	0.02	0.00	0.04
Sex (covariate)	0.19	0.08	2.44	0.02	0.04	0.34
Age (covariate)	−0.07	0.04	−1.68	0.09	−0.14	0.01
Overall model: *F*(10, 335) = 14.02, *p* = 0.00, *R*^2^ = 0.32						

^1^ T1 = pre-intervention and T2 = post-intervention.

## References

[1] World Health Organization (2016). Ending Childhood Obesity.

[2] PAHO/WHO, CARICOM (2006). Report of the Caribbean Commission on Health and Development.

[3] Schwiebbe L., van Rest J., Verhagen E., Visser R.W., Holthe J.K., Hira Sing R.A. (2011). Childhood obesity in the caribbean. West Indian Med. J..

[4] Visser R.W.M. (2008). Estado nutricional y perfil lipídico en escolares de 6 a 11 años en aruba. Rev. Cuba. Alim. Nutr..

[5] Government of Aruba, Special Committee on Obesity (2008). National Plan Aruba 2009–2018. For the Fight against Overweight, Obesity and Related Health Issues.

[6] Caribbean Public Health Agency (2015). Safeguarding Our Future Development. Plan of Action for Promoting Healthy Weights in the Caribbean: Prevention and Control of Childhood Obesity 2014–2019.

[7] Traboulay E.A., Hoyte O.P.A. (2015). Mini-review: Obesity in caribbean youth. West Indian Med. J..

[8] Greaux K., Schwiebbe L., Renders C.M., Doak C.M., Visser R., Holthe J.K., Hira Sing R.A. (2013). Breastfeeding and food pattern in overweight children in the caribbean. Paediatr. Int. Child Health.

[9] Department of Public Health Aruba (2013). Health Monitor Aruba 2013.

[10] Malik V.S., Pan A., Willett W.C., Hu F.B. (2013). Sugar-sweetened beverages and weight gain in children and adults: A systematic review and meta-analysis. Am. J. Clin. Nutr..

[11] Martin-Calvo N., Martínez-González M.-A., Bes-Rastrollo M., Gea A., Ochoa M.C., Marti A. (2014). Sugar-sweetened carbonated beverage consumption and childhood/adolescent obesity: A case-control study. Public Health Nutr..

[12] World Health Organization (2015). Guideline: Sugars Intake for Adults and Children.

[13] Singh G.M., Micha R., Khatibzadeh S., Shi P., Lim S., Andrews K.G., Engell R.E., Ezzati M., Mozaffarian D., NutriCoDE (2015). Global, regional, and national consumption of sugar-sweetened beverages, fruit juices, and milk: A systematic assessment of beverage intake in 187 countries. PLoS ONE.

[14] Tull E.S., Cort M.A., Taylor J., Wickramasuriya T. (2013). Understanding the relative influence of attitudes and societal norms on dietary intentions among african-caribbean women. Soc. Sci. J..

[15] Francis M., Nichols S.S.D., Dalrymple N. (2010). The effects of a school-based intervention programme on dietary intakes and physical activity among primary-school children in trinidad and tobago. Public Health Nutr..

[16] Caribbean Health Research Council (2011). Health Research Agenda for the Caribbean.

[17] Singh G.M., Micha R., Khatibzadeh S., Lim S., Ezzati M., Mozaffarian D. (2015). Estimated global, regional, and national disease burdens related to sugar-sweetened beverage consumption in 2010. Circulation.

[18] Vargas-Garcia E.J., Evans C.E.L., Prestwich A., Sykes-Muskett B.J., Hooson J., Cade J.E. (2017). Interventions to reduce consumption of sugar-sweetened beverages or increase water intake: Evidence from a systematic review and meta-analysis. Obes. Rev..

[19] Avery A., Bostock L., McCullough F. (2015). A systematic review investigating interventions that can help reduce consumption of sugar-sweetened beverages in children leading to changes in body fatness. J. Hum. Nutr. Diet..

[20] Smit C.R., Leeuw R.N.H.D., Bevelander K.E., Burk W.J., Buijzen M. (2016). A social network-based intervention stimulating peer influence on children’s self-reported water consumption: A randomized control trial. Appetite.

[21] Christakis N.A., Fowler J.H. (2011). Connected: The Amazing Power of Social Networks and How They Shape Our Lives.

[22] Valente T.W., Davis R.L. (1999). Accelerating the diffusion of innovations using opinion leaders. Ann. Am. Acad. Political Soc. Sci..

[23] Campbell R., Starkey F., Holliday J., Audrey S., Bloor M., Parry-Langdon N., Hughes R., Moore L. (2008). An informal school-based peer-led intervention for smoking prevention in adolescence (assist): A cluster randomised trial. Lancet.

[24] Salvy S.J., de la Haye K., Bowker J.C., Hermans R.C. (2012). Influence of peers and friends on children’s and adolescents’ eating and activity behaviors. Physiol. Behav..

[25] Cruwys T., Bevelander K.E., Hermans R.C.J. (2015). Social modeling of eating: A review of when and why social influence affects food intake and choice. Appetite.

[26] Herman C.P., Polivy J. (2005). Normative influences on food intake. Physiol. Behav..

[27] Higgs S. (2015). Social norms and their influence on eating behaviours. Appetite.

[28] Patrick H., Nicklas T.A. (2005). A review of family and social determinants of children’s eating patterns and diet quality. J. Am. Coll. Nutr..

[29] Pettigrew S., Jongenelis M., Chapman K., Miller C. (2015). Factors influencing the frequency of children’s consumption of soft drinks. Appetite.

[30] Rivis A., Sheeran P. (2003). Descriptive norms as an additional predictor in the theory of planned behaviour: A meta-analysis. Curr. Psychol..

[31] Robinson E., Thomas J., Aveyard P., Higgs S. (2014). What everyone else is eating: A systematic review and meta-analysis of the effect of informational eating norms on eating behavior. J. Acad. Nutr. Diet..

[32] Wouters E.J., Larsen J.K., Kremers S.P., Dagnelie P.C., Geenen R. (2010). Peer influence on snacking behavior in adolescence. Appetite.

[33] Cialdini R.B., Reno R.R., Kallgren C.A. (1990). A focus theory of normative conduct: Recycling the concept of norms to reduce littering in public places. J. Pers. Soc. Psychol..

[34] Starkey F., Audrey S., Holliday J., Moore L., Campbell R. (2009). Identifying influential young people to undertake effective peer-led health promotion: The example of a stop smoking in schools trial (assist). Health Educ. Res..

[35] Haerens L., Craeynest M., Deforche B., Maes L., Cardon G., De Bourdeaudhuij I. (2008). The contribution of psychosocial and home environmental factors in explaining eating behaviours in adolescents. Eur. J. Clin. Nutr..

[36] Kassem N.O., Lee J.W., Modeste N.N., Johnston P.K. (2003). Understanding soft drink consumption among female adolescents using the theory of planned behavior. Health Educ. Res..

[37] Van der Horst K., Kremers S., Ferreira I., Singh A., Oenema A., Brug J. (2007). Perceived parenting style and practices and the consumption of sugar-sweetened beverages by adolescents. Health Educ. Res..

[38] Bevelander K.E., Anschutz D.J., Engels R.C. (2012). Social norms in food intake among normal weight and overweight children. Appetite.

[39] Laerhoven H. (2004). A comparison of likert scale and visual analogue scales as response options in children’s questionnaires. Acta Paediatr..

[40] Hayes A.F. (2012). Process: A Versatile Computational Tool for Observed Variable Mediation, Moderation, and Conditional Process Modeling. http://www.afhayes.com/public/process2012.pdf.

[41] Driskell M.-M., Dyment S., Mauriello L., Castle P., Sherman K. (2008). Relationships among multiple behaviors for childhood and adolescent obesity prevention. Prev. Med..

[42] Nigg C.R., Burbank P.M., Padula C., Dufresne R., Rossi J.S., Velicer W.F., Laforge R.G., Prochaska J.O. (1999). Stages of change across ten health risk behaviors for older adults. Gerontologist.

[43] Hedrick V.E., Davy B.M., You W., Porter K.J., Estabrooks P.A., Zoellner J.M. (2017). Dietary quality changes in response to a sugar-sweetened beverage-reduction intervention: Results from the talking health randomized controlled clinical trial. Am. J. Clin. Nutr..

[44] Loughridge J.L., Barratt J. (2005). Does the provision of cooled filtered water in secondary school cafeterias increase water drinking and decrease the purchase of soft drinks?. J. Hum. Nutr. Diet..

[45] Mantziki K., Renders C.M., Seidell J.C. (2017). Water consumption in european children: Associations with intake of fruit juices, soft drinks and related parenting practices. Int. J. Environ. Res. Public Health.

[46] Mazarello Paes V., Hesketh K., O’Malley C., Moore H., Summerbell C., Griffin S., Sluijs E.M.F., Ong K.K., Lakshman R. (2015). Determinants of sugar-sweetened beverages in young children: A systematic review. Obes. Rev..

